# A randomized trial that compared brain activity, efficacy and plausibility of open-label placebo treatment and cognitive reappraisal for reducing emotional distress

**DOI:** 10.1038/s41598-023-39806-y

**Published:** 2023-08-26

**Authors:** Anne Schienle, Wolfgang Kogler, Albert Wabnegger

**Affiliations:** Department of Clinical Psychology, University of Graz, BioTechMed, Universitätsplatz 2/DG, 8010 Graz, Austria

**Keywords:** Neuroscience, Psychology

## Abstract

Placebo pills can reduce emotional distress even when recipients have been informed about the inert nature of the treatment. However, before such open-label placebos (OLPs) can be recommended for general clinical use, their efficacy and acceptability need to be further investigated and compared with established methods for emotion regulation, such as cognitive reappraisal (CR). The current study with functional magnetic resonance imaging compared the effects of an OLP pill with CR for reducing a specific form of emotional distress: disgust. Participants (150 healthy females) were randomly allocated to one of three groups, all of which were exposed to disgusting and neutral images (OLP, CR, PV: passive viewing). It was tested whether the three groups would differ in brain activity and reported disgust. Ratings for the perceived efficacy and plausibility of treatment were also compared between OLP and CR. Both OLP and CR increased the activity in a cognitive control region, the ventrolateral prefrontal cortex. Relative to PV and OLP, CR reduced activity in the putamen and pallidum. These regions play an important role in decoding disgust signals from different modalities. Self-reports indicated that CR was perceived as a more effective and plausible intervention strategy than OLP. In conclusion, CR was a superior method for disgust regulation compared to OLP, both on the subjective as well as the neurobiological level. Future OLP studies are needed to test whether the observed effects generalize to other forms of emotional distress.

## Introduction

Several neuroimaging studies have shown that placebos can reduce the intensity of negative affective states. For example, inert pills administered as ‘analgesics’, ‘anxiolytics’, or ‘anti-nausea medication’ can alleviate feelings of social rejection (emotional pain), anxiety, and disgust^1–4^. The effects of treatment with a deceptive placebo are seen not only in self-report measures but also in changes in activity of specific neural networks, including prefrontal cognitive control regions (e.g., ventrolateral/dorsolateral prefrontal cortex; VLPFC/DLPFC) and the insula/amygdala^1–4^. In sum, placebos can reduce both self-report and neural measures of emotional distress.

Despite these positive effects, the prescription of ‘deceptive placebos’ is surrounded by serious ethical issues. The lack of informed consent and transparency of treatment prohibits the clinical use of placebos. One approach that circumvents these ethical issues is the administration of open-label placebos (OLPs), which are presented to a person openly and honestly, without any deception regarding their nature^5^. The most widely used protocol for the administration of OLPs also entails information about placebo effects and the mechanisms assumed to be responsible for these effects (i.e., expectation, conditioning), coupled with a positive verbal suggestion: ‘The placebo effect is powerful’^6^.

The beneficial outcomes of OLP treatment for reducing emotional distress have been documented primarily using self-report measures. To the best of our knowledge, only three OLP studies have recorded neurobiological indicators of affective processing. In a functional magnetic resonance imaging (fMRI) study by Schaefer et al.^7^, participants received a placebo nasal spray to reduce negative emotional reactions, while looking at unpleasant images. The OLP treatment was associated with activation changes in the periaqueductal gray, the hippocampus, and the anterior cingulate cortex. These brain areas are involved in the modulation of affective states. In two experiments with electroencephalography (EEG), participants were also instructed that a placebo nasal spray may reduce visually induced emotional distress^8,9^. Both studies revealed that the OLP produced changes in a neural marker of emotion regulation, the late positive potential (LPP).

The LPP effects observed in the above-mentioned OLP studies (specifically, the reduction of frontal and centroparietal LPP amplitudes due to OLP treatment) shared striking similarities with findings from previous EEG experiments focusing on cognitive reappraisal [CR^10,11^]. CR refers to the cognitive reinterpreting of emotional stimuli. For instance, participants in reappraisal studies might internally remove themselves from the emotional context presented (such as by telling themselves that the images shown do not depict real situations), or they may intentionally evaluate the stimuli more positively (e.g., ‘This negative situation will turn out well’). CR is a well-established emotion regulation strategy, associated with various beneficial health outcomes^12^. Interestingly, it has been suggested that placebo effects are also mediated via affective (re)appraisal^13^ and that the placebo response can be understood as a ‘meaning response’ (i.e., that the placebo response is produced by the meaning found in a social setting such as a clinical encounter in which a pill is administered^14^.

A direct comparison of OLP treatment and CR could provide important insights into the underlying neural mechanisms of the two approaches. Functional magnetic resonance imaging (fMRI), a neurobiological method with excellent spatial resolution, presents itself as one effective approach for identifying brain networks involved in these interventions. It has been shown that the downregulation of negative emotional states using CR involves cognitive control regions in frontal areas of the brain (e.g., VLPFC/DLPFC). These areas modulate activity in (sub)cortical regions concerned with the decoding of stimulus salience (e.g., amygdala, insula; see meta-analysis^15^. For example, Goldin et al.^16^ presented participants with disgust-eliciting films. Relative to the passive viewing of these films, reappraisal decreased the negative emotion experience, which was accompanied by altered activity in the DLPFC, VLPFC, amygdala, and insula. To the best of our knowledge, there has been thus far no study carried out which has compared the effects of OLP and CR on emotional distress using fMRI.

Aside from establishing the effectiveness of OLPs on a subjective as well as neurobiological level, it is also important to investigate whether this type of intervention is found to be plausible by the treated individuals. Some recent studies have indicated that the concept of OLPs is not always perceived as intuitive by the placebo recipients [e.g.,^17,18^]. However, before being able to implement the OLP approach in clinical practice, it is important to take these aspects into account. Patients need to understand and accept a chosen intervention for symptom reduction to take place.

The current study with functional magnetic resonance imaging (fMRI) compared the effects of an OLP pill with CR for reducing a specific form of emotional distress: disgust. This basic emotion plays an important role in a wide range of psychopathology, including contamination-based obsessive–compulsive disorder, specific phobia (spider/blood), borderline personality disorder, and eating disorders^19^. Patients with these diagnoses often report intense and difficult-to-control disgust feelings when confronted with disorder-specific stimuli. To change these dysfunctional disgust processes, new treatment strategies are urgently needed because excessive symptoms of disgust are often resistant to conventional therapeutic approaches, such as CR^19^. However, individuals who experience difficulties in regulating disgust might profit from placebo interventions. In previous studies^4,20^, it has already been demonstrated that deceptive placebos can effectively reduce disgust feelings in healthy adults.

The present investigation explored whether an OLP can have similar disgust-reducing effects. The participants were randomly allocated to one of three groups (OLP, CR, PV: passive viewing), all of which were exposed to disgusting and neutral images. Ratings of disgust elicited by the pictures were assessed. It was hypothesized that participants of the OLP/CR groups would report less intense disgust feelings compared to the PV group. The reduced emotional distress should be accompanied by altered activity in prefrontal cognitive control regions (e.g., DLPFC, VLPFC, ACC) as demonstrated before in fMRI studies focusing on the effects of CR^16,20^, and placebo treatment^7,20^. Moreover, the CR and OLP interventions should change activity in the insula when compared with the passive viewing of the disgusting images^4,20^. Using an exploratory approach, it was investigated whether participants’ attitudes toward the treatment (perceived efficacy, plausibility) would differ between OLP and CR. Both groups were asked to rate the expected efficacy of the intervention (before the picture viewing) as well as the perceived efficacy (after the picture viewing). Finally, CR/OLP participants evaluated the plausibility of the treatment rationale.

## Results

### Disgust ratings

The computed multilevel-model analysis of variance (mlmANOVA) revealed a significant main effect of GROUP (F(2,147) = 14.27, *p* < 0.001), PICTURE CATEGORY (F(1,2847) = 7196.38, *p* < 0.001), as well as a significant interaction GROUP X PICTURE CATEGORY (F(2,2847) = 62.39, *p* < 0.001). Bonferroni-corrected post-hoc t-tests showed that relative to the PV group, the OLP group (mean difference = 0.54, t = 3.17, *p* = 0.028, d = 0.39) and the CR group (mean difference = 1.48, t = 8.63, *p* < 0.001, d = 1.02) reported less intense disgust. The CR group gave lower disgust ratings than the OLP group (mean difference = 0.94, t = 5.46, *p* < 0.001, d = 0.63; Table 1).

**Table 1:** Self-reports (means, standard deviations) of the three groups.

Overall	Open-label placebo	Cognitive reappraisal	Passive viewing
(SD)	(SD)	(SD)	(SD)
[95% CI]	[95% CI]	[95% CI]	[95% CI]
Picture ratings: disgust intensity [1..9]			
Disgust			
5.21 (1.5)	5.34 (1.45)	4.40 (1.54)	5.88 (1.34)
[4.96, 5.46]	[4.91, 5.72]	[4.00, 4.86]	[5.52, 6.25]
Neutral			
1.28 (0.45)	1.27 (0.41)	1.17 (0.34)	1.41 (0.55)
[1.21, 1,37]	[1.17, 1.38]	[1.08, 1.26]	[1.26, 1.57]
Questionnaires			
Disgust Propensity			
2.57 (0.48)	2.61 (0.56)	2.58 (0.41)	2.51 (0.48)
[2.49, 2.65]	[2.46, 2.77]	[2.46, 2.68]	[2.38, 2.65]
Reappraisal			
4.98 (0.91)	5.00 (0.96)	4.96 (0.93)	4.97 (0.87)
[4.83, 5.12]	[4.74, 5.27]	[4.71, 5.19]	[4.71, 5.20]

### Efficacy and plausibility of CR and OLP

The mlmANOVA indicated significant main effects of GROUP (F(1,97) = 42.45, *p* < 0.001), and TIME (F(1,96.5) = 5.90, *p* = 0.017), and a significant interaction GROUP X TIME (F(1,96.5) = 5.90, *p* = 0.017) for the efficacy ratings. Post-hoc tests with Bonferroni correction showed that relative to the OLP group, the CR group gave higher ratings for expected efficacy (CR M = 6.46, SD = 1.16; OLP: M = 5.10, SD = 1.66; t = 4.29, *p* < 0.001, d = 0.95) and perceived efficacy of the intervention (CR M = 6.46, SD = 1.63; OLP: M = 4.27, SD = 1.81; t = 6.81, *p* < 0.001, d = 1.27). In the OLP group, the perceived efficacy after the picture viewing was lower than the expected efficacy (difference: M = − 0.81, t = 3.42 *p* = 0.005, d = 0.46). Expected versus perceived efficacy did not differ from each other (difference: M = 0) in the CR group.

Ratings for the plausibility of the treatment rationale differed between the two groups with a higher value in the CR group (M = 7.68, SD = 1.29) compared to the OLP group (M = 6.32, SD = 2.26, t(98) = -3.70, *p* < 0.001, d = 0.74). Plausibility ratings were positively correlated with the efficacy ratings for the interventions (OLP/expected efficacy: r = 0.37, *p* = 0.008; perceived efficacy: r = 0.25, *p* = 0.08; CR/expected efficacy: r = 0.26, *p* = 0.06; perceived efficacy: r = 0.36, *p* = 0.009).

The percentages of participants who gave efficacy/plausibility ratings for the interventions at medium or higher levels (≥ 5; center of the scale) were as follows: expected efficacy: 70% (OLP) versus 92% (CR), perceived efficacy: 49% (OLP) versus 90% (CR); plausibility: 72% (OLP) versus 98% (CR).

### Brain activity

**Figure 1:**
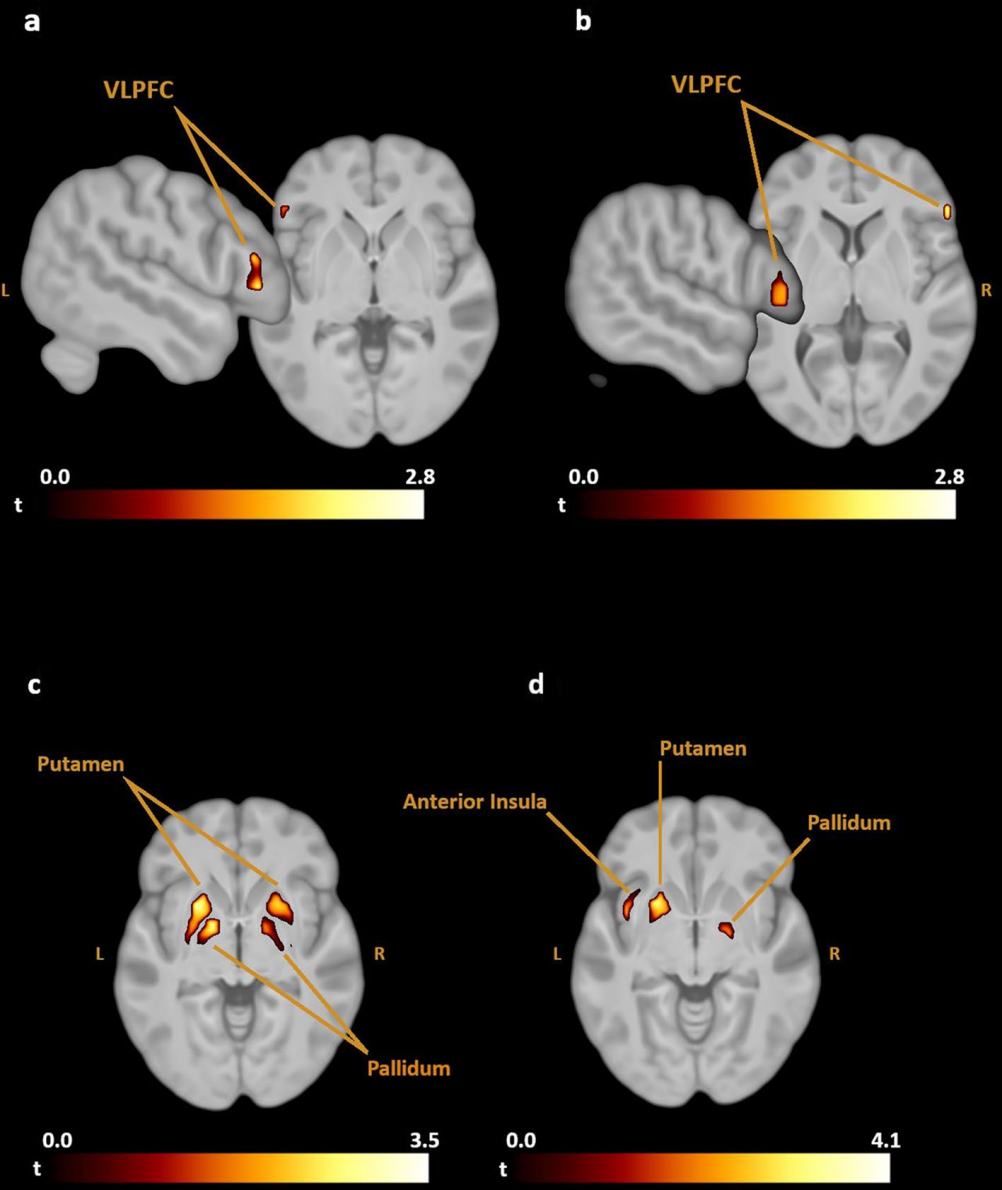
Differences in activation between (**a**) cognitive reappraisal—passive viewing, (**b**) open-label placebo—passive viewing, (**c**) open-label placebo—cognitive reappraisal, (**d**) passive viewing—cognitive reappraisal for the contrast disgust—neutral. *VLPFC* ventrolateral prefrontal cortex.

Region-of-interest analyses: The group comparisons for the contrast Disgust > Neutral indicated that both cognitive reappraisal (CR) and the open-label placebo (OLP) were associated with increased activity in the ventrolateral prefrontal cortex (VLPFC) relative to the passive viewing (PV) of the disgust pictures (corresponding Brodmann areas were BA 47 for the contrast CR-PV and BA 45 for the contrast OLP-PV). CR decreased activity in the putamen, pallidum, and anterior insula compared to PV. The direct comparison of OLP with CR indicated greater activity in the putamen and pallidum for the placebo group (see Fig. 1, Table 2). Within-group findings are displayed in Supplementary Table S1. Statistically significant whole-brain findings were not detected.

**Table 2:** Group comparison of brain activity (contrast: disgust > neutral).

Region of interest	H	MNI coordinates					
		X	y	z	t	p_FWE-corr	Cluster size
Cognitive reappraisal—passive viewing							
Ventrolateral prefrontal cortex	L	− 49	33	− 1	2.72	0.0429	60
Open-label placebo—passive viewing							
Ventrolateral prefrontal cortex	R	56	30	4	2.72	0.0322	51
Passive viewing—cognitive reappraisal							
Putamen	L	− 24	13	− 9	3.24	0.0406	125
Pallidum	R	19	− 8	− 6	3.48	0.0089	34
Anterior insula	L	− 39	10	− 14	3.08	0.0383	52
Open label placebo—cognitive reappraisal							
Pallidum	L	− 14	3	− 4	4.00	0.0019	83
Pallidum	R	14	− 3	− 6	3.22	0.0182	44
Putamen	L	− 24	13	− 6	3.94	0.0057	242
Putamen	R	24	13	− 9	3.64	0.0154	154
Passive viewing—open-label placebo							
No significant findings							
Cognitive Reappraisal—open-label placebo							
No significant findings							

## Discussion

Research has demonstrated that OLPs can reduce emotional distress^7–9^. However, before the use of OLPs can be generally recommended, their efficacy and acceptability need to be compared with established methods for emotion regulation (e.g., CR cognitive reappraisal). Moreover, the underlying neural mechanisms of OLPs are thus far poorly understood. To fill this knowledge gap, the present study compared two approaches (CR, OLP) for regulating visually induced emotional distress (disgust), based on self-reports and neuroimaging data from participants (healthy females). Both intervention groups were also compared with another group that engaged in passive-picture viewing (PV).

It was found that the CR group and the OLP group gave lower disgust ratings for the negative pictures compared to the PV group. While CR had a large effect on disgust reduction (difference CR vs. PV), the effect of the OLP was medium-sized (OLP vs. PV). Similar changes in self-reports for emotional distress have been reported before in studies on cognitive reappraisal^16,20,21^ and OLPs^8,9,22,23^.

The neuroimaging data indicated the involvement of the ventrolateral prefrontal cortex (VLPFC) during both CR and OLP treatment compared to the passive viewing of the disgusting pictures. The VLPFC has been found to be activated during the use of various strategies for voluntary emotion regulation, such as distraction, expression inhibition, and CR (see meta-analysis by^24^). A meta-analysis that specifically focused on CR studies^15^ also documented the pivotal role of the VLPFC in the context of reappraisal (i.e., of changing one's interpretations of affective stimuli). The authors of that study suggested that the VLPFC may support the selection and inhibition of appraisals (i.e., the judgment of what could be a suitable reappraisal or not). More specifically, Cheng et al.^25^ have proposed that the left and right VLPFC may have distinct roles in emotion regulation. They concluded that the left VLPFC is responsible for linguistic-semantic processes of generating and selecting appraisals. This interpretation fits nicely with the findings of the present study with enhanced left VLPFC recruitment during CR (contrast: CR > PV). Some participants indicated in their commentaries after the experiments that they had rehearsed the CR instruction during the picture viewing (‘the shown situations and objects are not real’).

Participants of the OLP group displayed increased activity in the right VLPFC (contrast: OLP > PV). The right VLPFC plays a critical role in motor inhibition and (visual) attention^26^. Previous studies on CR and deceptive placebos using eye-tracking have demonstrated that both approaches differ in attention direction. Using CR for down-regulating negative affect (implemented by imagining that the situation in the picture is not real) was associated with attentional deployment away from emotional areas within an aversive image^27^. In contrast, the administration of a deceptive placebo enhanced the number of fixations for unpleasant images^28^. Thus, the placebo facilitated attentional engagement. Future research is needed to directly compare the attentional processes that characterize CR and OLP interventions. This is especially important because the direct contrast of left/right VLPFC activity between the CR and OLP groups did not reveal statistically significant results. Thus, based on the current data it would be premature to assume lateralized VLPFC effects in CR and OLP treatment.

The passive viewing of the disgusting images was associated with activity in the putamen and pallidum. This replicates previous findings of several fMRI studies that used visual disgust elicitors^29–31^. The putamen is involved in recognizing signals of disgust in others, as well as being closely linked to the experience of this basic emotion^29^. Further, the pallidum has been shown to play a role in initiating (disgust-related) avoidance behavior. In the present study, both the PV group and the OLP group showed greater putamen/pallidum activity than the CR group. Moreover, OLP and PV did not differ in putamen/pallidum activity. These findings indicate that the placebo was not able to reduce neural correlates of disgust feelings as effectively as cognitive reappraisal.

In line with this interpretation is the result concerning insula recruitment, which was more pronounced during PV relative to CR (contrast: PV > CR). The insula is part of a salience network that plays an important role in tracking emotions associated with bodily states, such as disgust, and generally for the representation of interoceptive processes^32^.

To sum up the fMRI findings it can be stated that CR increased activation in a cognitive control region (VLPFC) and reduced activation in regions that decode the salience and punishment value of stimuli (insula, putamen/pallidum). The OLP treatment also recruited the VLPFC but was not able to lower activation in the putamen, pallidum, and insula.

The observed activation differences between OLP and CR imply that the placebo was a less efficient method for reducing disgust. This interpretation is also supported by participants’ efficacy ratings for the treatments. Both the expected efficacy before the picture viewing as well as the perceived efficacy after the picture viewing were higher in the CR group compared to the OLP group. This effect was large as indicated by the associated effect size measures. Further, the OLP group gave lower efficacy ratings after the picture viewing compared to before the picture viewing, whereas the ratings given by the CR group remained temporally stable. This effect might be interpreted as a sign of disappointment concerning the OLP intervention (which appeared for the participants to not work out as well as expected). A similar finding has been reported previously in a study that also administered an OLP for reducing emotional distress induced by negative pictures^9^; at the end of this study, participants also indicated that the OLP did not completely meet their expectations.

Finally, CR was rated as a more plausible strategy for disgust regulation than the OLP. Participants of the CR group indicated few problems in following the instructions. In contrast, some participants in the OLP group reported that they found the idea to swallow a sugar pill for reducing emotional distress to be unconvincing. In both groups (CR, OLP), ratings for the plausibility of the rationale were positively associated with the expected and perceived effectiveness of the intervention.

Although the current study found OLP to be an intervention that was on average less effective than CR, half of the participants (49%) reported profiting from the non-deceptive placebo treatment (and gave efficacy ratings ≥ 5 after the picture viewing). Therefore, future studies need to identify those factors (personal, contextual) that are associated with the reactivity to OLP treatment.

Some limitations of the current study should be mentioned. First, our sample included only female participants with a limited age range and educational background (with the majority being students). Therefore, our findings may not apply to other sociodemographic groups. Second, this study focused on disgust processing. Therefore, the present findings cannot be generalized to other forms of emotional distress. For example, a previous OLP study with fMRI^7^ used images with different negative contents (e.g., accidents, illness, violence) to induce emotional distress. At the end of the experiment, the participants rated how negative the pictures made them feel (without referring to specific emotions). The identified OLP-reactive brain regions (ACC, hippocampus, periaqueductal gray) differ from the present study. This may hint at emotion-specific OLP effects (e.g., fear vs. disgust), which should be the focus of future investigations. Benedetti et al.^33^ have already pointed out that there is no single placebo effect but many, depending on the specific conditions being treated in specific contexts.

In sum, the current study showed CR to be a superior method for disgust regulation in healthy female participants compared to OLP, on both the subjective as well as the neurobiological level.

## Materials and methods

### Participants

A total of 150 females (mean age = 23.5 years, SD = 5.24; 90% university students) participated in this study. They were recruited via announcements at the university campus and through social media. Inclusion criteria were a minimum age of 18 years and female sex. We investigated a female sample because of gender differences in placebo reactivity^34^ and disgust propensity (the temporally stable tendency of a person to experience disgust across different situations;^35^). Exclusion criteria were reported diagnoses of mental disorders, neurological disorders, intake of psychotropic medication, and contraindications for MRI scans (e.g., pregnancy, metal implants).

The three groups did not differ in mean age (*F*(2,147) = 0.24, *p* = 0.79), scores on the brief Questionnaire for the Assessment of Disgust Propensity^34^; F(2,147) = 0.54, *p* = 0.58) and on the reappraisal scale of the Emotion Regulation Questionnaire^36^ 2003; F(2,147) = 0.036, *p* = 0.97; see Table 1).

A power analysis conducted with G*Power^37^ showed that for three groups and two picture types (Disgust, Neutral) and an effect size of 0.16 (partial eta^2^ = 0.03) together with an alpha error probability of 0.05 and a power of 0.95, 150 participants would be needed.

### Experimental Design and Procedure

The study was conducted at the University of Graz (Austria) and complied with all relevant ethical guidelines and regulations involving human participants and was approved by the ethics committee of the University of Graz (GZ. 39/64/63 ex 2021/22). All participants provided informed consent before participating. This study was preregistered on the German Clinical Trials Register (DRKS00029250, 08/06/2022) and conducted between June and September 2022. The CONSORT diagram is depicted in Supplementary Figure S1.

The participants were invited to an fMRI study on emotional processing (no information about placebos was provided in the invitation). They were randomly assigned (random number table) to one of three groups: The Open-Label Placebo group (OLP; *n* = 50), the Cognitive Reappraisal group (CR *n* = 50), and the Passive Viewing group (PV; *n* = 50). One experimenter generated the random allocation sequence and assigned participants to the interventions. Two other experimenters conducted the experiment.

Each group received different instructions before the picture viewing task via a power-point presentation (14 slides with text and illustrations; no audio; fixed timing with 30 s per slide). The presentations followed the procedure of previous studies^8,9^ and were comparable between groups in terms of the number of presented slides, figures, and words. The OLP group viewed a presentation about the neurobiological effects of placebos with a focus on affective processing (findings from fMRI/EEG studies). Subsequently, they received a white 1 cm long capsule filled with 0.8 g dextrose labeled as a “placebo capsule”. The participants of the OLP group were informed that the placebo contains sugar but may help them to reduce their disgust feelings in the subsequent picture-viewing task.

For the CR group, the presentation provided information about the neurobiological effects of emotion regulation/cognitive reappraisal (findings from fMRI/EEG studies). After the presentation, the participants were instructed to apply the strategy of cognitive reappraisal in the picture viewing task by imagining that the shown situations and objects are not real, but created by a special effects artist for a Halloween movie. This instruction has been successfully used before^20,27^.

The PV group viewed a presentation with information on affective neuroscience (findings from fMRI/EEG studies). Afterward, the participants received the same capsule as the OLP group. The capsule was introduced as an “MRI capsule”. It was stated that the capsule is filled with dextrose to enhance the metabolism-associated signal-to-noise ratio in the MRI (improvement of measurement). After the study participants rated the plausibility of this explanation on a scale ranging from 1 to 9 (very plausible). The average rating was *M* = 7.00 (*SD* = 2.16).

After the instructions, the OLP group and the CR group evaluated their expectations concerning the effectiveness of the intervention (“What do you think? How effective will the OLP/cognitive reappraisal be in reducing your negative emotional responses to the images?” 1 = not effective; 9 = very effective). Then, the participants were brought to the MRI scanner in the adjacent room. Here, the group-specific instructions were summarized (OLP: “Please remember that the placebo you received may help you to reduce your negative emotional reactions to the images”; CR: “Please remember that the objects and situations shown in the pictures are not real, but have been created by a special effects artist”; PV: “Please remember to watch each image carefully for the entire duration of the presentation”).

After the picture viewing, participants in the OLP group and the CR group were asked to rate the perceived effectiveness of the intervention (1 = not effective; 9 = very effective) and the plausibility of the rationale (1 = not logical; 9 = very logical).

### Picture viewing task

The participants viewed 30 disgusting and 30 neutral images. The disgust pictures depicted core disgust elicitors (e.g., disgusting animals such as snails and worms, rotten food, and body secretions) and were taken from the International Affective Picture System^38^, and other validated picture sets^20^. The neutral images consisted of pixelated versions of the disgust images with a mosaic-like appearance (same color, luminance). These images also had been validated in a previous study^20^.

The pictures were presented within blocks of three pictures of the same type (10 disgust blocks, 10 neutral blocks). The sequence of blocks was randomized, with the only restriction that a maximum of two blocks of the same type could be shown consecutively. Within a block, the pictures were presented for 5 s each. Then, a fixation cross appeared for a variable interval (2–4 s), which was followed by a rating for experienced disgust (“How strong was your experienced disgust?”) on a scale ranging from 1 to 9 (very strong). Participants gave the rating verbally using the intercom system. The picture presentation only continued when the rating was completed. Before the next block started, a fixation cross was presented for 15 s (indicating a resting period).

### fMRI recording and analysis

The MRI session was conducted with a 3T scanner (Vida, Siemens, Erlangen, Germany) with a 64-channel head coil. Functional runs were acquired using a T2*-weighted multiband EPI protocol (number of slices: 58, interleaved, flip angle = 82°, slice thickness: 2.5 mm; slice spacing: 2.5 mm; TE = 0.03; TR = 1800 ms; multi-band accel. factor = 2; acquisition matrix: 88; in-plane resolution = 2.5 × 2.5 × 2.5 mm). Structural images were obtained using a T1-weighted MPRAGE sequence (voxel size: 1 × 1 × 1 mm; acquisition matrix: 224, slice thickness: 1 mm, TE = 0.00236, TR = 1600 ms; flip angle = 9°). Preprocessing of the fMRI data was performed using *fMRIPrep* 22.0.1^39^; RRID: SCR_016216), which is based on *Nipype* 1.8.4^40^; RRID: SCR_002502; see Supplementary Material S2). Smoothing and first/second level analyses were carried out with SPM12 (7487) implemented in Matlab R2019b.

#### First-level analysis

Before conducting the first-level analyses, functional image quality metrics were evaluated using MRIQC^41^. Substantial deviations in various quality-related metrics, such as signal-to-noise ratio and outliers, led to the exclusion of five participants (OLP: 2; PV: 1; CR 2) from further analyses. The preprocessed images were then used for the first-level analyses, which involved convolving event-related responses for the image categories (Disgust, Neutral), and rating scales with the hemodynamic response function. We defined the contrast of interest as Disgust minus Neutral. To account for motion-induced variance, we used the six motion parameters and their first derivative as regressors of no interest. Volumes with a framewise displacement exceeding the predefined threshold of 0.5 mm were excluded from further analyses. The data were high-pass filtered at a frequency of 180 s, and serial correlations were addressed by using an autoregressive AR(1) model.

#### Second-level analysis

We compared the contrast of interest (Disgust—Neutral) between groups (CR vs. OLP; CR vs. PV; OLP vs. PV). Regions of interest (ROIs) were selected based on previous research^7,19^ and included the ventrolateral prefrontal cortex (VLPFC), dorsolateral prefrontal cortex (DLPFC), anterior cingulate cortex (ACC), insula, putamen, and pallidum. The ROI masks were derived from the Harvard–Oxford probability atlas (with a threshold of 50%). Given the absence of explicit masks for the DLPFC and VLPFC in the Harvard–Oxford atlas, we used the inferior frontal gyrus as the VLPFC mask^42^, while the middle and superior frontal gyrus formed the mask for the DLPFC^43^. Statistical significance was determined based on a family-wise corrected (FWE) *p*-value for voxel peaks that was below 0.05.

### Statistical analysis of self-report data

A multilevel-model analysis of variance (mlmANOVA) was used to compare disgust ratings between GROUPs (CR, OLP, PV) and PICTURE CATEGORIES (Disgust, Neutral). MlmANOVAs offer several advantages over repeated-measures ANOVAs, such as dealing with missing values or when the number of repeats varies. Furthermore, mlmANOVAs explicitly model random effects due to the clustering (observations nested within a cluster (e.g., subjects), resulting in cluster-specific coefficients for the intercept and/or slope. For the current analyses, we defined subjects as the cluster variable and allowed the intercept to vary. The effects of GROUP (CR, OLP) and TIME (before vs. after the picture viewing) on expected/perceived effectiveness of the treatment were also examined using a multilevel-model ANOVA with subjects as the cluster variable (varying intercepts). We used the GAMLj package of JAMOVI (version: 2.3.21).

Bonferroni-corrected t-tests were calculated for post-hoc analysis following significant main effects/interactions of the multilevel-model ANOVAs. A t-test was conducted to compare the plausibility of the treatment rationale between CR and OLP. We report Cohen’s d as an effect size measure.

### Supplementary Information

Supplementary Information.

## Data Availability

The datasets used and/or analyzed during the current study are available from the corresponding author upon reasonable request.
