# Author Correction: A randomized trial that compared brain activity, efficacy and plausibility of open-label placebo treatment and cognitive reappraisal for reducing emotional distress

**DOI:** 10.1038/s41598-023-49408-3

**Published:** 2023-12-19

**Authors:** Anne Schienle, Wolfgang Kogler, Albert Wabnegger

**Affiliations:** Department of Clinical Psychology, University of Graz, BioTechMed, Universitätsplatz 2/DG, 8010 Graz, Austria

Correction to: *Scientific Reports* 10.1038/s41598-023-39806-y, published online 26 August 2023

The Acknowledgement section in the original version of this Article was omitted. The Acknowledgement section now reads:

“The authors acknowledge the financial support from the University of Graz.”

The original Article has been corrected.

