# Supplementary material for: A randomized trial that compared brain activity, efficacy and plausibility of open-label placebo treatment and cognitive reappraisal for reducing emotional distress
Source: Sci Rep. 2023 Aug 26;13:13998. doi: 10.1038/s41598-023-39806-y (PMC10460441; doi:10.1038/s41598-023-39806-y)
Supplement: Supplementary file 1 — Supplementary Information. [file 41598_2023_39806_MOESM1_ESM.pdf]

Supplementary Table S1: Brain activity (contrast: Disgust > Neutral) within the three groups

ROI	H	X	Y	Z	T	p_FWE-corr	Cluster Size
**Passive Viewing**							
Insula	L	-37	5	-14	11.81	p<.001	217
Insula	R	36	10	-16	12.30	p<.001	238
Pallidum	L	-22	-10	-6	10.08	p<.001	113
Pallidum	R	14	-5	-6	10.71	p<.001	113
Putamen	L	-29	-13	-11	12.94	p<.001	253
Putamen	R	19	5	-11	11.02	p<.001	300
Ventrolateral prefrontal cortex	L	-49	33	12	6.21	p<.001	65
Ventrolateral prefrontal cortex	R	54	25	2	5.06	p<.001	40
Dorsolateral prefrontal cortex	L	-17	33	54	8.50	p<.001	502
Dorsolateral prefrontal cortex	R	6	13	59	9.58	p<.001	502
Anterior cingulate cortex	L	-4	28	27	9.74	p<.001	744
**Open Label Placebo**							
Insula	L	-39	8	-6	11.88	p<.001	211
Insula	R	36	8	-14	11.21	p<.001	255
Pallidum	L	-17	-8	-6	10.50	p<.001	118
Pallidum	R	14	-3	-6	8.62	p<.001	117
Putamen	L	-29	-13	-11	11.53	p<.001	341
Putamen	R	19	5	-11	12.27	p<.001	395
Ventrolateral prefrontal cortex	L	-52	30	17	6.31	p<.001	80
Ventrolateral prefrontal cortex	R	54	33	12	7.02	p<.001	51
Dorsolateral prefrontal cortex	L	-14	3	67	10.13	p<.001	320
Dorsolateral prefrontal cortex	R	4	13	59	9.80	p<.001	320
Anterior cingulate cortex	R	4	18	37	10.26	p<.001	730
**Cognitive Reappraisal**							
Insula	L	-34	8	-14	10.81	p<.001	206
Insula	R	36	10	-14	10.66	p<.001	214
Pallidum	L	-19	-10	-6	9.87	p<.001	118
Pallidum	R	21	-10	-6	8.85	p<.001	81
Putamen	L	-29	-13	-11	16.91	p<.001	301
Putamen	R	31	-10	-11	8.47	p<.001	272
Ventrolateral prefrontal cortex	L	-52	33	14	9.13	p<.001	80
Ventrolateral prefrontal cortex	R	54	30	7	7.84	p<.001	51
Dorsolateral prefrontal cortex	L	-4	15	54	11.92	p<.001	563
Dorsolateral prefrontal cortex	R	4	15	57	9.91	p<.001	563
Anterior cingulate cortex	R	4	3	32	9.94	p<.001	624

Supplementary Figure S1: CONSORT flow diagram

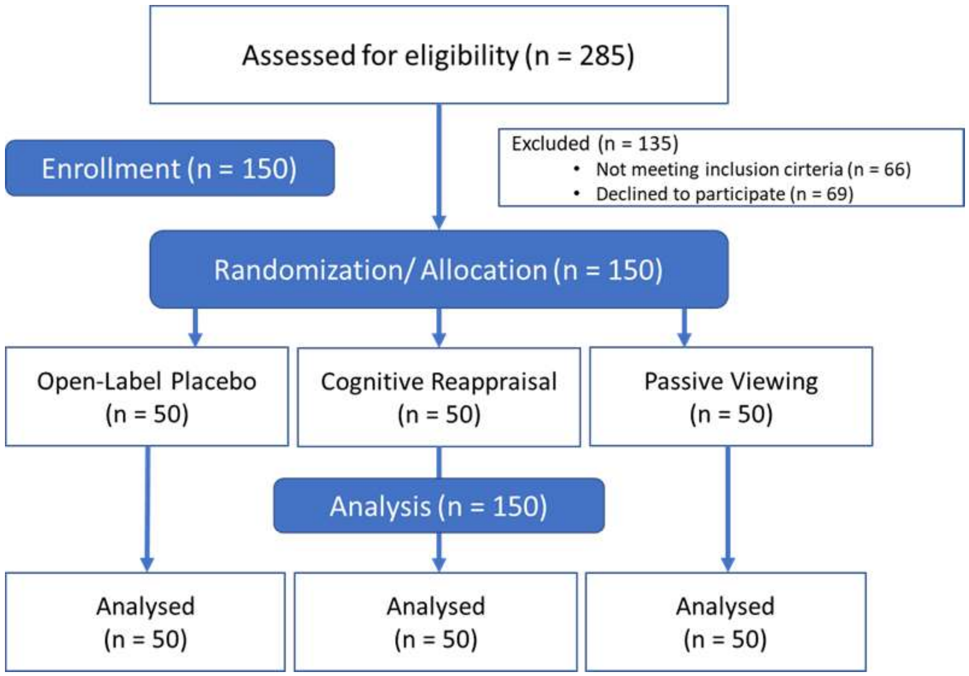

Supplementary Table S2: Preprocessing of fMRI data

Preprocessing of B_0 inhomogeneity mappings
A B0-nonuniformity map (or fieldmap) was estimated based on two (or more) echo-planar imaging (EPI) references with topup (Andersson, Skare, and Ashburner (2003); FSL 6.0.5.1:57b01774).
Preprocessing of anatomical data
The T1-weighted (T1w) image was corrected for intensity non-uniformity (INU) with N4BiasFieldCorrection (Tustison et al. 2010), distributed with ANTs 2.3.3 (Avants et al. 2008, RRID:SCR_004757), and used as T1w-reference throughout the workflow. The T1w-reference was then skull-stripped with a *Nipype* implementation of the antsBrainExtraction.sh workflow (from ANTs), using OASIS30ANTs as target template. Brain tissue segmentation of cerebrospinal fluid (CSF), white-matter (WM) and gray-matter (GM) was performed on the brain-extracted T1w using fast (FSL 6.0.5.1:57b01774, RRID:SCR_002823, Zhang, Brady, and Smith 2001). Volume-based spatial normalization to one standard space (MNI152NLin2009cAsym) was performed through nonlinear registration with antsRegistration (ANTs 2.3.3), using brain-extracted versions of both T1w reference and the T1w template. The following template was selected for spatial normalization: *ICBM 152 Nonlinear Asymmetrical template version 2009c* [Fonov et al. (2009), RRID:SCR_008796; TemplateFlow ID: MNI152NLin2009cAsym].
Preprocessing of functional data
For each of the 1 BOLD run found per subject (across all tasks and sessions), the following preprocessing was performed. First, a reference volume and its skull-stripped

version were generated using a custom methodology of *fMRIPrep*. Head-motion parameters with respect to the BOLD reference (transformation matrices, and six corresponding rotation and translation parameters) are estimated before any spatiotemporal filtering using mcflirt (FSL 6.0.5.1:57b01774, Jenkinson et al. 2002). The estimated *fieldmap* was then aligned with rigid-registration to the target EPI (echo-planar imaging) reference run. The field coefficients were mapped on to the reference EPI using the transform. BOLD runs were slice-time corrected to 0.859s (0.5 of slice acquisition range 0s-1.72s) using 3dTshift from AFNI (Cox and Hyde 1997, RRID:SCR_005927). The BOLD reference was then co-registered to the T1w reference using mri_coreg (FreeSurfer) followed by flirt (FSL 6.0.5.1:57b01774, Jenkinson and Smith 2001) with the boundary-based registration (Greve and Fischl 2009) cost-function. Co-registration was configured with six degrees of freedom. Several confounding time-series were calculated based on the *preprocessed BOLD*: framewise displacement (FD), DVARS and three region-wise global signals. FD was computed using two formulations following Power (absolute sum of relative motions, Power et al. (2014)) and Jenkinson (relative root mean square displacement between affines, Jenkinson et al. (2002)). FD and DVARS are calculated for each functional run, both using their implementations in *Nipype* (following the definitions by Power et al. 2014). The three global signals are extracted within the CSF, the WM, and the whole-brain masks. Additionally, a set of physiological regressors were extracted to allow for component-based noise correction (*CompCor*, Behzadi et al. 2007). Principal components are estimated after high-pass filtering the *preprocessed BOLD* time-series (using a discrete cosine filter with 128s cut-off) for the two *CompCor* variants: temporal (tCompCor) and anatomical (aCompCor). tCompCor components are then calculated from the top 2% variable voxels within the brain mask. For aCompCor, three probabilistic masks (CSF, WM and combined CSF+WM) are generated in anatomical

space. The implementation differs from that of Behzadi et al. in that instead of eroding the masks by 2 pixels on BOLD space, a mask of pixels that likely contain a volume fraction of GM is subtracted from the aCompCor masks. This mask is obtained by thresholding the corresponding partial volume map at 0.05, and it ensures components are not extracted from voxels containing a minimal fraction of GM. Finally, these masks are resampled into BOLD space and binarized by thresholding at 0.99 (as in the original implementation). Components are also calculated separately within the WM and CSF masks. For each CompCor decomposition, the *k* components with the largest singular values are retained, such that the retained components' time series are sufficient to explain 50 percent of variance across the nuisance mask (CSF, WM, combined, or temporal). The remaining components are dropped from consideration. The head-motion estimates calculated in the correction step were also placed within the corresponding confounds file. The confound time series derived from head motion estimates and global signals were expanded with the inclusion of temporal derivatives and quadratic terms for each (Satterthwaite et al. 2013). Frames that exceeded a threshold of 0.5 mm FD or 1.5 standardized DVARS were annotated as motion outliers. Additional nuisance timeseries are calculated by means of principal components analysis of the signal found within a thin band (*crown*) of voxels around the edge of the brain, as proposed by (Patriat, Reynolds, and Birn 2017). The BOLD time-series were resampled into standard space, generating a *preprocessed BOLD run in MNI152NLin2009cAsym space*. First, a reference volume and its skull-stripped version were generated using a custom methodology of *fMRIPrep*. All resamplings can be performed with *a single interpolation step* by composing all the pertinent transformations (i.e. head-motion transform matrices, susceptibility distortion correction when available, and co-registrations to anatomical and output spaces). Gridded (volumetric) resamplings were performed using antsApplyTransforms (ANTs),

configured with Lanczos interpolation to minimize the smoothing effects of other kernels (Lanczos 1964). Non-gridded (surface) resamplings were performed using mri_vol2surf (FreeSurfer). Finally, functional images were smoothed with a Gaussian full width at half maximum of 8mm.
